# Unique Duplication of *trnN* in *Odontoptilum angulatum* (Lepidoptera: Pyrginae) and Phylogeny within Hesperiidae

**DOI:** 10.3390/insects12040348

**Published:** 2021-04-14

**Authors:** Jiaqi Liu, Jintian Xiao, Xiangyu Hao, Xiangqun Yuan

**Affiliations:** 1 Key Laboratory of Plant Protection Resources and Pest Management, Ministry of Education, Entomological Museum, College of Plant Protection, Northwest A&F University, Yangling 712100, China; jiaq_work@163.com (J.L.); xjt0629@nwafu.edu.cn (J.X.); 2 College of Life Sciences, Northwest A&F University, Yangling 712100, China; xyhao@nwsuaf.edu.cn

**Keywords:** Tagiadini, mitogenome, TDRL model, tRNA gene duplication, phylogenetic analysis

## Abstract

**Simple Summary:**

Pyrginae is one of the major groups of the Hesperiidae, and has some particular characteristics. The annotated complete mitogenome from this subfamily is reported here. The gene order of the new mitogenome with the duplication of *trnN* differs from the typical Lepidoptera-specific arrangement and is unique to Hesperiidae. The presence of a pseudo gene in the mt genome of *Odontoptilum angulatum* supports the duplication of *trnN*, following the TDRL model. Comparison of the newly generated mitogenome of *Odontoptilum angulatum* to all available mitochondrial genomes of other rearranged Pyrginae species revealed that the condition of *Odontoptilum angulatum* is of independent origin. Therefore, we hypothesize that the gene block *trnN*–*trnS1*–*trnE* is the hot spot of gene rearrangement in the Tagiadini of Pyrginae. Phylogenetic analyses based on 13 protein-coding genes and entire RNA genes of mitogenomes show the monophyly of Pyrginae.

**Abstract:**

To explore the variation and relationship between gene rearrangement and phylogenetic effectiveness of mitogenomes among lineages of the diversification of the tribe Tagiadini in the subfamily Pyrginae, we sequenced the complete mitogenome of *Odontoptilum angulatum*. The genome is 15,361 bp with the typical 37 genes, a large AT-rich region and an additional *trnN* (*trnN2*), which is completely identical to *trnN* (sequence similarity: 100%). The gene order differs from the typical Lepidoptera-specific arrangement and is unique to Hesperiidae. The presence of a “pseudo-*trnS1*” in the non-coding region between *trnN1* and *trnN2* supports the hypothesis that the presence of an extra *trnN* can be explained by the tandem duplication-random loss (TDRL) model. Regarding the phylogenetic analyses, we found that the dataset comprising all 37 genes produced the highest node support, as well as a monophyly of Pyrginae, indicating that the inclusion of RNAs improves the phylogenetic signal. Relationships among the subfamilies in Hesperiidae were also in general agreement with the results of previous studies. The monophyly of Tagiadini is strongly supported. Our study provides a new orientation for application of compositional and mutational biases of mitogenomes in phylogenetic analysis of Tagiadini and even all Hesperiidae based on larger taxon sampling in the future.

## 1. Introduction

Due to unique characteristics of the insect mitochondrion, such as strict maternal mode of inheritance, conservative gene components and a comparatively fast rate of evolution [1,2,3], mitochondrial genomes have become a significant molecular marker due to their maternal inheritance, fast evolutionary rate and highly conserved gene content compared to nuclear genes [4], and have been used widely in studies of genetic evolution, classification and identification, and indicators of possible phylogenic relationships for many taxonomic groups including Lepidopteran insects [5,6,7]. Generally, the insect mitogenome, which contains 13 protein-coding genes (PCGs), 2 ribosomal RNA genes (rRNAs), 22 transfer RNA genes (tRNAs) and a non-coding region of variable length, is a typical covalently closed circular double-stranded DNA molecule [2,3,8]. In recent years, with improvements in next-generation sequencers, lower cost and increased availability of whole mitogenome data [3,9], the complete sequences of mitogenomes are being applied more widely in inferring phylogenetic relationships and the molecular evolution of mitogenomes [3,10].

The arrangement of genes in most insect mitogenomes is highly conservative. This can be explained by the compact arrangement of genes, the short non-coding sequences between genes, and the frequent overlap of genes consisting of a few nucleotides [11,12,13]. However, gene rearrangements (especially in tRNA genes [2]) are commonly reported in some taxa (such as Thysanoptera [14,15,16], Phthiraptera [17,18], and Hymenoptera [19]). Furthermore, more exotic rearrangements of the mitochondrial genome have been reported, such as its fragmentation into multiple circular microchromomes (for example, the South Asia 1 species of *Scirtothrips dorsalis* has a genome consisting of two circular chromosomes [20]). This gene movement provides more information in phylogenetic reconstruction [3]. In Lepidoptera, *trnM* was translocated upstream of *trnI* (*trnM*-*trnI*-*trnQ*) in the species of Ditrysia, which is consistent with results from the first Lepidopteran species (Bombyx mori) sequenced, whereas this gene cluster maintained the order of the ancestral gene (*trnI*-*trnQ*-*trnM*) in non-Ditrysian lineages [11]. However, the primitive taxa in Lepidoptera still need more sequencing for in-depth study of evolution and phylogeny.

The current mechanisms used to explain mitochondrial gene rearrangement include: tandem duplication-random loss (TDRL) [21], tandem duplication-nonrandom loss (TDNR) [22], recombination [23,24,25], and illicit priming of replication by tRNA genes [26]. The TDRL model is the most widely accepted mechanism in order to explain the occurrence of gene rearrangement, duplicated genes in mitogenomes, and the occurrence of novel gene order of duplicated genes [27,28,29]. According to the TDRL model, slipped-strand mispairing, imprecise termination, dimerization of the genome, or recombination causes the duplication of a tandem segment of genes [21]. Subsequently, the accumulation of mutations in multiple gene duplications will eventually cause one of the genes to lose function. At this time, such selective pressure to shrink the genome will lead to the elimination of non-functional genes [30].

The family Hesperiidae is one of the most species-rich groups in butterflies, accounting for one-fifth of the world’s butterfly species [31]. Pyrginae is a subfamily of Hesperiidae, which includes 646 species in 86 genera worldwide [32,33,34,35,36]. Because of their natural beauty, such as in the *Odontoptilum*, it has become an important insect group for ornamental use and as a craft resource [37]. The mitogenomes of 6 Pyrginae species representing 6 genera have been sequenced, and only 2 tRNA duplications and/or tRNA pseudo genes are recorded in the subfamily Pyrginae. Both of them belong to the tribe Tagiadini, which are 2 of the only 3 available mitogenomes for the tribe: *Ctenoptilum vasava* (*trnS1* duplication and *trnL2* pseudo gene) [38] and *Tagiades vajuna* (a tandem duplication of *trnS1* and *trnE*) [39]. However, the remaining member, *Tagiades* (=*Daimio*) *tethys*, possesses a standard mitogenome. Therefore, it is speculated that this characteristic (tRNA duplication) may be is an autapomorphy in the tribe Tagiadini. To explore this phenomenon further, the mt genome of *Odontoptilum angulatum* was sequenced. Aunique gene arrangement with a duplicated *trnN* (*trnN1*-*trnN2*-*trnS1*) was detected for the first time in the subfamily Pyrginae, which is even unique among Lepidoptera. The presence of a pseudo *trnS1* and an upstream 7 bp gene fragment provided evidence that the TDRL model could explain this novel gene order.

## 2. Materials and Methods

### 2.1. Sample Collection and DNA Extraction

Two female adults of *O. angulatum* were collected in Longmen Village, Mengla County, Xishuangbanna Dai Autonomous Prefecture, Yunnan Province, China (21°815′ N; 101°29′ E). The entire samples were immediately put into 100% ethanol and, on returning to the lab, stored in a −20 °C freezer in the Entomological Museum of the Northwest A&F University, Yangling, Shaanxi Province, China. All adult specimens were identified based on morphological characteristics [37] and were confirmed with *cox1* barcoding alignment in the BOLD database [40]. The thoracic muscle from a single specimen was used to extract genomic DNA following the manufacturer’s instructions (EasyPureR Genomic DNA Kit, TRAN, TransGen, Beijing, China).

### 2.2. Sequencing

Complete mitochondrial genome was determined using NGS by Biomarker Technologies Corporation (Beijing, China.). After the samples were sequenced in both directions, approximately 1 GB of data was obtained. The raw paired-end clean reads were assembled under medium-low sensitivity with trim sequence by employing the mitogenome of *Tagiades vajuna* (Hesperiidae; Pyrginae; GenBank: KX865091) [39] as a reference (Table 1) in Geneious 11.0.5. The generated consensus sequence and the reference sequence were aligned used MAFFT (within Geneious 11.0.5) for annotation. All PCGs were determined by finding the ORFs by employing condon Table 5 (the invertebrate mitochondrial genetic code). The tRNAs, including the duplicated tRNA (*trnN*), and rRNAs (12S and 16S) were found using the MITOS Web Server [41]. According to the MITOS predictions, the secondary structures of tRNAs were manually plotted using Adobe Illustrator CC2020. The mitogenomic circular map was drawn using Organellar Genome DRAW (OGDRAW) [42]. Finally, in order to ensure the accuracy of annotation, all genes were visually inspected via alignments in Geneious against the reference mitogenome. The base composition, AT skew, CG skew and data used to plot RSCU (relative synonymous codon usage) figures were all calculated using PhyloSuite v 1.2.2 [43]. The mitogenome sequence was registered in GenBank as MW381783.

### 2.3. Phylogenetic Analysis

For the phylogenetic analysis, we chose a total of 33 mitogenome sequences of known Hesperiidae that are publicly available (including the newly sequenced mt genome of *O. angulatum*) (Table 1) as ingroups and the mitogenome of four species in Papilionidae were selected as outgroups.

Statistics for the basic characteristics of the mitochondrial genome were produced by MitoTool [62]. The extraction of PCGs and RNAs were carried out by PhyloSuite v 1.2.2. All 13 PCGs were aligned in batches with MAFFT integrated into BioSuite [63], based on the codon-alignment mode. tRNAs and rRNAs were aligned using the Q-INS-i algorithm in MAFFT v.7 online service [64]. Poorly aligned regions in the alignments were removed using Gblocks v0.91b [65].

To compare the phylogenetic signal information of the different dataset combinations, 3 datasets were used: protein-coding genes (PCGs), removal of third codon position of protein-coding genes + whole RNA genes (PCG12RT), and protein-coding genes + entire RNA genes (PCGRT). We chose the GTR + I + G model of evolution based on the three datasets by Bayesian information criterion (BIC) as estimated in jModelTest 2.1.7 [66]. Phylogenetic analysis was performed employing the best-fit model, using maximum likelihood (ML) and Bayesian inference (BI). The ML analysis were conducted using RAxML GUI [67], with an ML + rapid bootstrap (BS) algorithm with 1000 replicates. The BI analysis was implemented using MrBayes 3.2.6 [68] with default settings and 6 × 10^6^ MCMC generations. The convergence of the independent runs was indicated by average standard deviation of split frequencies < 0.01, estimated sample size > 200, and potential scale reduction factor ≈ 1.

## 3. Results and Discussion

### 3.1. Mitochondrial Genome Organization

**Figure 1:**
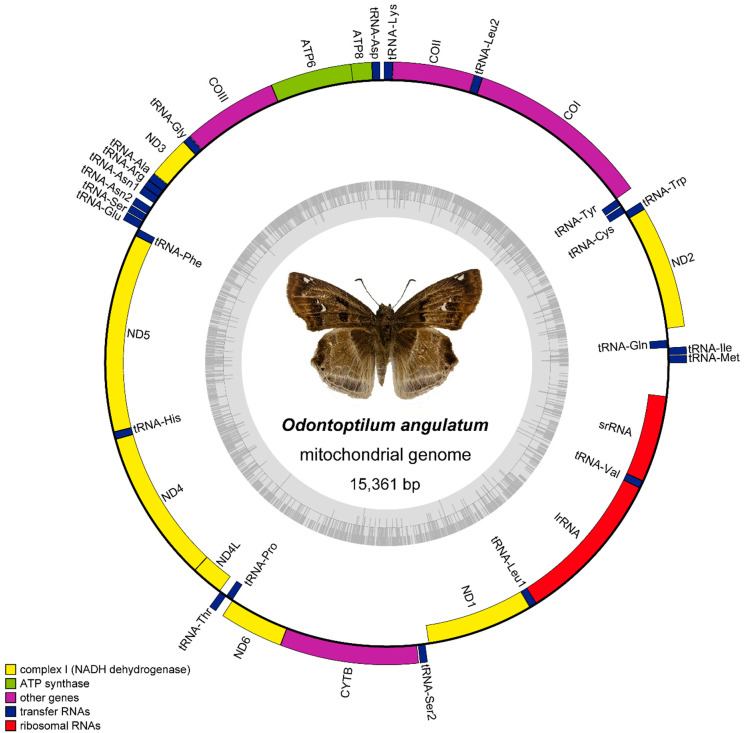
Circular map of the mitochondrial genome of *O. angulatum*.

The mitogenome of *O. angulatum* (15,361 bp) is a single, covalently closed circular double-stranded DNA molecule (Figure 1) composed of 38 coding genes (13 PCGs, 23 tRNA genes, and two rRNA genes), and a major non-coding A + T-rich region (replication origin site) [69]. Compared with the mitogenomes of other Hesperiidae (range from 15,267 bp (*Potanthus flavus*) to 15,769 bp (*Heteropterus morpheus*)), it is medium-sized. In addition to the 37 typical genes in arthropod mitochondrion, an additional *trnN* (named *trnN2*) was found in this genome.

The minority strand (N-strand) encodes 14 genes, 4 PCGs (*ND1*, *ND4*, *ND4L*, and *ND5*), 8 tRNAs (*trnQ*, *trnC*, *trnY*, *trnF*, *trnH*, *trnP*, *trnL2*, and *trnV*), and 2 rRNA genes (the large rRNA subunit (*lrRNA*) and small rRNA subunit (*srRNA*)), whereas the other 24 genes were transcribed from the majority strand (J-strand). All 13 PCGs were initiated by the start codon ATN (8 by ATG, 4 by ATT and 1 by ATA). Nine PCGs ended with a complete TAA termination codon. It is worth mentioning that, differing from the use of CGA in *cox1* initiation as has been reported before for Hesperiidae [70], *cox1* of *O. angulatum* starts with normal ATG. In addition to *cox1*, *cox2*, *nad5* and *nad4*, all of which use an incomplete stop codon T–, the remaining PCGs all use complete TAA stop codons. Seven gene overlaps were observed, ranging from 1 to 24 bp in length. With the exception of the control region, we identified 17 non-coding regions (NCRs) comprising a total of 261 bp with the second longest being 44 bp between *trnN1* and *trnN2*. The *lrRNA* and *srRNA* were 1380 bp and 761 bp, respectively (Table 2).

**Figure 2:**
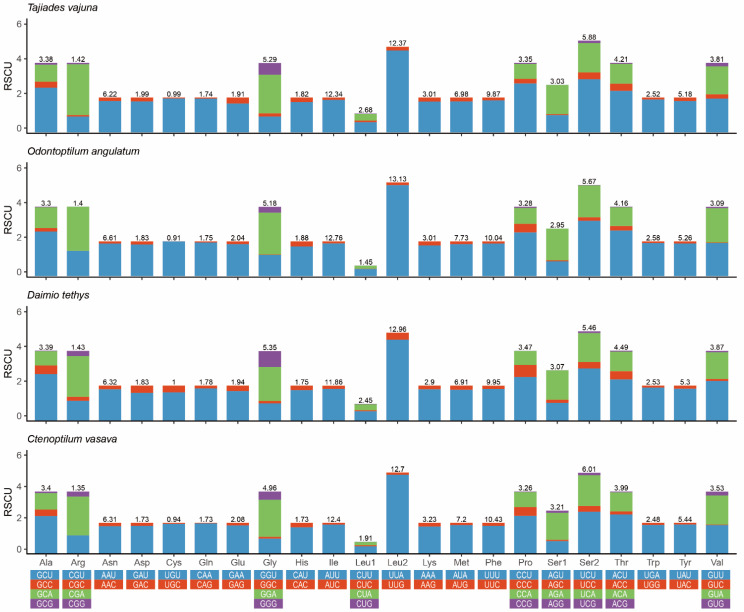
Relative synonymous codon usage (RSCU) in the mitochondrial genome of four species of Tagiadini. The stop codon is not shown.

The A/T nucleotide composition is 81.2% (excluding the control region) in *O. angulatum*, indicating a strong A/T bias (Table 3). The PCGs have the lowest AT content (79.8%) and the A + T-rich region has the highest (95.4%), as in all previously-sequenced mitogenomes of skippers [57]. Among the 13 PCGs, the A + T content of the third codon (94%) was much higher than the first (74.8%) and second positions (70.7%). The mitogenome exhibits obvious negative GC-skews (−0.217) and insignificant negative AT-skews (−0.015) (Table 3). The mitogenome-wide AT bias was well-documented in the codon usage, and the RSCU (relative synonymous codon usage) values indicated a preference for NNU and NNA codons in skipper mitogenomes, which has been observed before [38,45]. Furthermore, Figure 2 also indicates that the most frequently used codons are UUU (Phe), UUA (Leu), AUU (Ile), AUA (Met), and AAU (Asn).

In the whole genome of *O. angulatum*, besides the common 22 tRNAs, an additional *trnN* was found. We found a unique rearrangement that differs from previous studies in Lepidoptera [71,72]. Moreover, the sequences of *trnN1* and *trnN2* were absolutely identical (sequence similarity: 100%). Twenty-three tRNA (62 bp to 71 bp) could be folded into the typical structure of cloverleaf secondary (Figure S1). Only *trnS* (AGN) lacked the DHU stem (Figure S1), and this phenomenon probably evolved very early in the Metazoa [73] and is the ancestral state in butterflies. The *trnN1* and *trnN2* have a secondary structure of standard tRNA genes [74,75] and a completely identical anticodon; we surmise that both of them had identical functions.

### 3.2. Non-Coding Regions (NCR) and a Pseudo Gene

**Figure 3:**
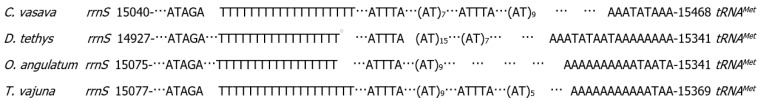
Structural elements found in the A + T-rich region of four Tagiadini skippers.

**Figure 4:**
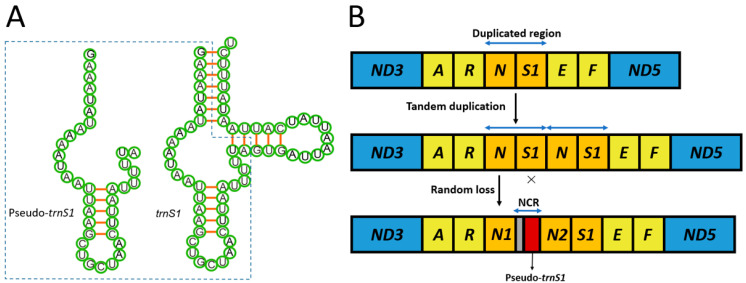
(**A**) Secondary structures and sequence similarity of *trnS1* and pseudo-*trnS1*. Inferred Watson–Crick bonds are illustrated by red lines. The sequences in the dotted box represent the homologous sequences between *trnS1* and pseudo-*trnS1*. (**B**) The hypothetical process of gene rearrangement in the model of tandem duplication-random loss. “×” indicates the partial random loss of the duplicated genes. Different types of genes are labeled with different colored blocks: PCGs-blue, tRNAs-yellow, pseudo gene-red and NCR-grey.

The control region, located between *rrnS* and *trnM* and thought to play a controlling role in the transcription process [76], was the longest non-coding region in the mitogenome of *O. angulatum*. Its length and A + T content are 287 bp and 95.4%, respectively (Table 3). Compared with three other skippers from Tagiadini, the sequence analysis result of the control region showed that they all have conserved structures, including variable-length poly-T stretches (17–19 bp), several runs of microsatellite-like A/T sequences following a motif ATTTA, and an interrupted poly-A stretch directly upstream of *trnM*. Moreover, the motif ATAGA close to the 5′-end of the *rrnS* is the origin of the minority strand replication in Lepidopteran mitogenomes (Figure 3) [69,77]. In addition to the control region, another long NCR (44 bp) was found between *trnN1* and *trnN2*. In this NCR, a 37 bp region was identified, which shares 100% similarity with the homologous sequences of *trnS1*. Therefore, we defined this region as pseudo-*trnS1* (Figure 4A). In addition, we found that the pseudo-*trnS1* had a 7 bp gene fragment (ATAATAT), which is the intergenic nucleotides between *trnN2* and *trnS1* in front of it in NCR.

The tandem duplication-random loss (TDRL) model is the most widely accepted mechanism for explaining mitochondrial gene rearrangement. In the TDRL model, some of the mitochondrial genes produce multiple gene repeats due to a contiguous segment of DNA duplication. Subsequently, the accumulation of mutations within multiple duplications eventually deactivates one of the genes at random, and the selective pressure to shrink the genome leads to the elimination of nonfunctional genes [30]. In this process, the randomly lost and nonfunctional genes can become pseudo genes. [78,79]. Pseudo genes may eventually disappear completely from the genome due to the selective pressure of shrinking the genome, causing the gene order to be different from the typical gene arrangement (such as *Drosophila yakuba*) [80].

In a previous study, the extra tRNA genes were also found in other Tagiadini species. The *trnS1* duplication in *Ctenoptilum vasava* (Hesperiidae: Pyrginae) (-N-S1a-S1b-E-) [38] and the tandem duplication of the gene block *trnS1*-*trnE* in *Tagiades vajuna* (Hesperiidae: Pyrginae) (-N-S1a -Ea -S1b -Eb -) [39] further indicates the independent origin of the duplicated *trnS1*: -N-S1-E- → -N-S1a-S1b-E- in *C. vasava*, -N-S1-E- → -N-S1a -Ea -S1b -Eb - in *Tagiades vajuna* and -N-S1-E- → -Na-Nb-S1-E- in *O. angulatum*. Therefore, the condition of *O. angulatum* is of independent origin. However, pseudo genes were not found in the vicinity of duplicated tRNA in both cases. The gene order of the *O. angulatum* mitogenome differs from the typical Lepidoptera-specific arrangement and is unique not only in Hesperiidae but also in Lepidoptera (Figure 1). The presence of pseudo-*trnS1* and an upstream 7 bp gene fragment in the mitogenome of *O. angulatum* supported the hypothesis that duplication of *trnN* obeyed the TDRL model. This current genomic rearrangement likely occurred due to the tandem duplication of the gene block *trnN*–*trnS1*, forming the structure of *trnN*–*trnS1*–*trnN*–*trnS1* and then the subsequent random loss of *trnS1* in the first copy, causing the current arrangement *trnNa*–*trnNb*–*trnS1* and a non-coding region including a 37-bp pseudo-*trnS1* (Figure 4B). The translocation of *trnN* and *trnS1* in the *E. montanus* (Pyrginae: Erynnini) mitogenome could also be explained through the TDRL model through a *trnN*–*trnS1* tandem duplication [51]. Therefore, we hypothesize that the gene block *trnN*–*trnS1*–*trnE* is the hot spot of gene rearrangement in the Tagiadini of Pyrginae.

### 3.3. Phylogenetic Analyses

**Figure 5:**
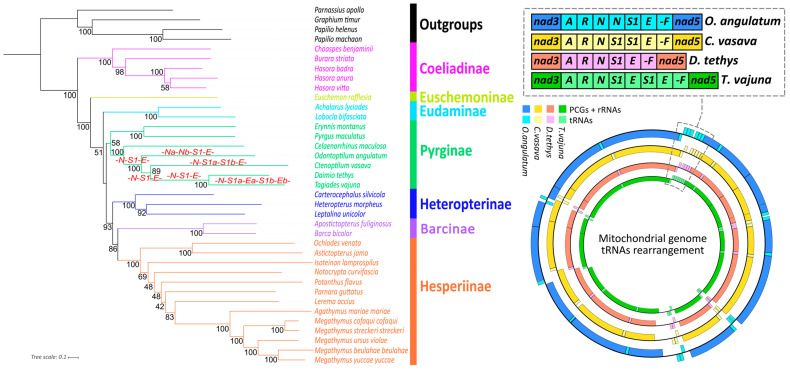
Phylogenetic tree produced by maximum likelihood analyses of PCGRT dataset. Bootstrap support values (BS) are indicated above the branches. The different gene orders of Tagiadini species are shown on the right of the tree.

The six phylogenetic trees (3 datasets × 2 methods) show topologies that are nearly congruent with most branches receiving strong support. The topologies generated by the PCGRT dataset have a higher support rate than PCG and PCG12RT, which indicates that the RNA genes have more phylogenetic resolution [81]. Since the phylogenetic topologies obtained by both methods (ML and BI) using the PCGRT dataset are concordant, we only showed the ML tree (Figure 5, all remaining dendrograms are shown in supplementary data: PCGRT dataset with the method of the BI in Figure S2, PCG dataset with the method of the ML and BI in Figures S3 and S4, and PCG12RT dataset with the method of ML and BI in Figures S5 and S6).

In general, the phylogenomic relationships recovered in our analysis are nearly identical to the most recent mitochondrial phylogenomic studies [39,54,57]. Nevertheless, Pyrginae and Eudaminae show different phylogenetic relationships in the 6 phylograms: Pyrginae was polyphyletic by Eudaminae in both analyses of the PCG datase and PCG12RT datasets, but monophyletic in the BI and ML analysis of the PCGRT dataset (nodal support value: BS = 58, BPP = 0.796). Nowadays, based on the study of the mitogenome data and united mitogenome/nuclear genome datasets, the monophyly of Pyrginae still remains unclear [82]. For the sake of resolving the problem of the taxonomic status of Pyrginae, a denser taxonomic sampling of mt genomes, more complete transcriptome or genomics data, and better linkage between morphological features and molecular data is required.

As expected, *O. angulatum* clustered with the other three Tagiadini species, *T. tethys*, *C. vasava*, *T. tethys* and *T. vajuna* in all 6 phylograms produced. Moreover, a consistent topology was obtained: (((*T. tethys* + *T. vajuna*) + *C. vasava*) + *O. angulatum*). The gene rearrangement in mitogenomes was expected to provide valuable information for the reconstruction of molecular phylogeny. However, it seems that majority of gene rearrangements could be observed in the tribe Tagiadini within the family Hesperiidae. Theoretically, it is beneficial to use mt genome rearrangement as a phylogenetic marker because rearrangements of the mt genome appear to be unique and rare events, which are stable once they have occurred, and the rearrangement genes are homologous. The synapomorphy of gene rearrangement supported an insect–crustacean clade and further study of the arrangements of the mt genome will help to understand and to improve the higher-level taxonomy and systematics [83,84].

## 4. Conclusions

We sequenced the mitogenome of *O. angulatum* and found that the duplication of *trnN* was unique among all the characterized mitogenomes in Lepidoptera. The duplication of *trnN* obeyed the TDRL model, where one of the *trnS1* lost some parts and became a pseudo-gene after the tandem-duplication of the element *trnN1*-*trnS1*. Comparing with the duplication pattern of closely related branch, we suggest that the *trnN* duplication of *O. angulatum* probably has an independent origin in Tagiadini. We compared the results from different datasets and methods to reconstruct the phylogenetic reconstruction of the family Hesperiidae, and suggest that the topologies generated by the PCGRT dataset had a higher node support rate.

## Figures and Tables

**Table 1:** Classification and origins of the mitochondrial genome used in this study.

Taxon	Species	Accession Number	References
Hesperiidae			
Coeliadinae	*Burara striata*	NC_034676	[44]
	*Choaspes benjaminii*	NC_024647	[45]
	*Hasora anura*	KF881049	[46]
	*Hasora vitta*	NC_027170	[47]
	*Hasora badra*	NC_045249	Unpublished
Euschemoninae	*Euschemon rafflesia*	NC_034231	[48]
Pyrginae	*Celaenorrhinus maculosus*	NC_022853	[49]
	*Ctenoptilum vasava*	JF713818	[38]
	*Tagiades (*=*Daimio) tethys*	KJ813807	[50]
	*Erynnis montanus*	NC_021427	[51]
	*Pyrgus maculatus*	NC_030192	Unpublished
	*Tagiades vajuna*	KX865091	[39]
	*Odontoptilum angulatum*	MW381783	This study
Eudaminae	*Achalarus lyciades*	NC_030602	[52]
	*Lobocla bifasciata*	KJ629166	[45]
Heteropterinae	*Carterocephalus silvicola*	NC_024646	[45]
	*Heteropterus morpheus*	NC_028506	Unpublished
	*Leptalina unicolour*	MK265705	[53]
Barcinae	*Apostictopterus fuliginosus*	NC_039946	[54]
	*Barca bicolor*	NC_039947	[54]
Hesperiinae	*Lerema accius*	NC_029826	[55]
	*Ochlodes venata*	HM243593	Unpublished
	*Parnara guttata*	NC_029136	[56]
	*Potanthus flavus*	KJ629167	[45]
	*Astictopterus jama*	MH763663	[57]
	*Isoteinon lamprospilus*	MH763664	[57]
	*Notocrypta curvifascia*	MH763665	[57]
	*Agathymus mariae*	KY630504	[58]
	*Megathymus beulahae*	KY630505	[58]
	*Megathymus cofaqui*	KY630503	[58]
	*Megathymus streckeri*	KY630501	[58]
	*Megathymus ursus*	KY630502	[58]
	*Megathymus yuccae*	KY630500	[58]
Outgroup			
Papilionidae	*Papilio machaon*	NC_018047	Unpublished
	*Papilio helenus*	NC_025757	[59]
	*Graphium timur*	NC_024098	[60]
	*Parnassius apollo*	NC_024727	[61]

**Table 2:** Mitogenomic organization of *O. angulatum*.

	Position		Size (bp)	Intergenic Nucleotides	Codon		Strand
	From	To			Start	Stop	
*trnM*	1	66	66				+
*trnI*	72	135	64	5			+
*trnQ*	133	201	69	−3			-
*nad2*	300	1313	1014	98	ATT	TAA	+
*trnW*	1312	1378	67	−2			+
*trnC*	1371	1435	65	−8			-
*trnY*	1446	1510	65	10			-
*cox1*	1513	3046	1534	2	ATG	T	+
*trnL2*	3047	3113	67				+
*cox2*	3115	3793	679	1	ATG	T	+
*trnK*	3794	3864	71				+
*trnD*	3898	3963	66	33			+
*atp8*	3964	4137	174		ATT	TAA	+
*atp6*	4131	4808	678	−7	ATG	TAA	+
*cox3*	4808	5593	786	−1	ATG	TAA	+
*trnG*	5596	5662	67	2			+
*nad3*	5663	6016	354		ATT	TAA	+
*trnA*	6019	6084	66	2			+
*trnR*	6085	6148	64				+
*trnN1*	6149	6214	66				+
*trnN2*	6259	6324	66	44			+
*trnS1*	6332	6393	62	7			+
*trnE*	6399	6469	71	5			+
*trnF*	6473	6536	64	3			+
*nad5*	6537	8280	1744				-
*trnH*	8281	8345	65		ATA	T	-
*nad4*	8346	9684	1339				-
*nad4L*	9685	9966	282		ATG	T	-
*trnT*	9974	10,036	63	7	ATG	TAA	-
*trnP*	10,037	10,100	64				+
*nad6*	10,103	10,633	531	2			-
*cytb*	10,633	11,781	1149	−1	ATT	TAA	+
*trnS2*	11,791	11,854	64	9	ATG	TAA	+
*nad1*	11,885	12,823	939	30			+
*trnL1*	12,825	12,891	67	1	ATG	TAA	-
*rrnL*	12,868	14,247	1380	−24			-
*trnV*	14,248	14,313	66				-
*rrnS*	14,314	15,074	761				-
A-T rich region	15,075	15,361	287				+

**Table 3:** Nucleotide composition and skewness of *O. angulatum*.

*O. angulatum*								
Regions	Size (bp)	T(U)	C	A	G	AT (%)	AT Skew	GC Skew
PCGs	11199	46.1	9.9	33.7	10.2	79.8	−0.155	0.014
1st codon position	3733	37.7	9.7	37.1	15.4	74.8	−0.008	0.226
2nd codon position	3733	48.1	16.4	22.6	13	70.7	−0.361	−0.115
3rd codon position	3733	52.5	3.8	41.5	2.3	94	−0.117	−0.239
A + T rich region	287	47	2.8	48.4	1.7	95.4	0.015	−0.231
tRNAs	1515	39.4	7.7	42	10.9	81.4	0.032	0.174
rRNAs	2141	41.3	5.0	43.7	10	85	0.027	0.34
Full genome	15361	41.2	11.4	40	7.3	81.2	−0.015	−0.217

## Data Availability

The following information was supplied regarding the availability of DNA sequences: The complete mitogenome of *Odontoptilum angulatum* is deposited in GenBank of NCBI under accession number MW381783.

## Supplementary Materials

The following are available online at https://www.mdpi.com/article/10.3390/insects12040348/s1, Figure S1: Predicted secondary cloverleaf structure for the tRNAs of *O. angulatum*. Lines (-) indicate Watson–Crick base pairings, whereas dots (·) indicate unmatched base pairings; Figure S2: Phylogenetic tree produced by Bayesian inference analysis of the PCGRT dataset. Bayesian posterior probability (BPP) support values are indicated above the branches; Figure S3: Phylogenetic tree produced by maximum likelihood analyses of PCG dataset. Bootstrap support values (BS) are indicated above the branches; Figure S4: Phylogenetic tree produced by Bayesian inference analysis of the PCG dataset. Bayesian posterior probability (BPP) support values are indicated above the branches; Figure S5: Phylogenetic tree produced by maximum likelihood analyses of PCG12RT dataset. Bootstrap support values (BS) are indicated above the branches; Figure S6: Phylogenetic tree produced by Bayesian inference analysis of the PCG12RT dataset. Bayesian posterior probability (BPP) support values are indicated above the branches.
