# Supplementary figures and images for: Unique Duplication of trnN in Odontoptilum angulatum (Lepidoptera: Pyrginae) and Phylogeny within Hesperiidae
Source: Insects. 2021 Apr 14;12(4):348. doi: 10.3390/insects12040348 (PMC8070526; doi:10.3390/insects12040348)

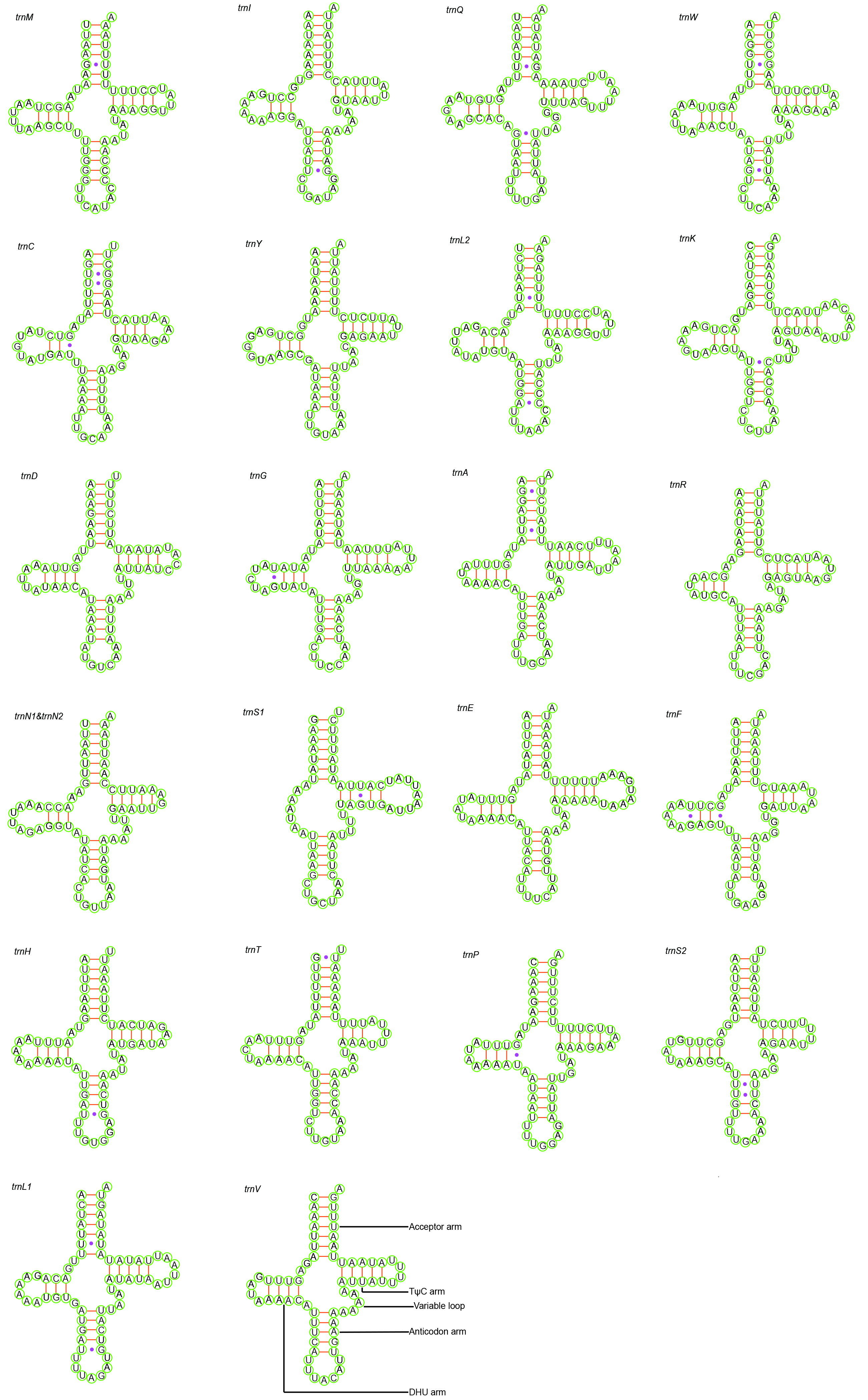

Supplement: Supplementary file 1 [file insects-12-00348-s001.zip › Fig. S1.tif]

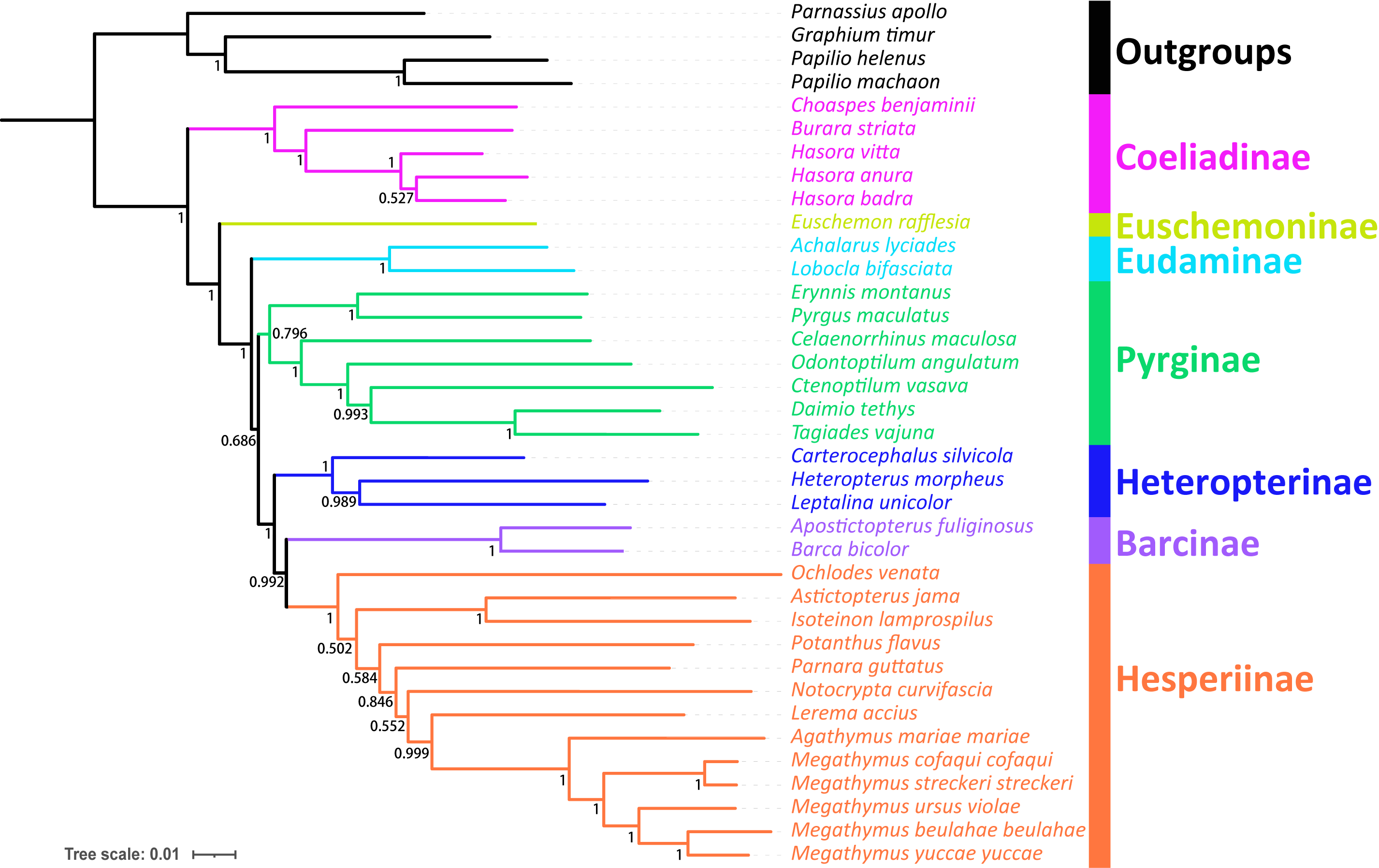

Supplement: Supplementary file 1 [file insects-12-00348-s001.zip › Fig. S2.tif]

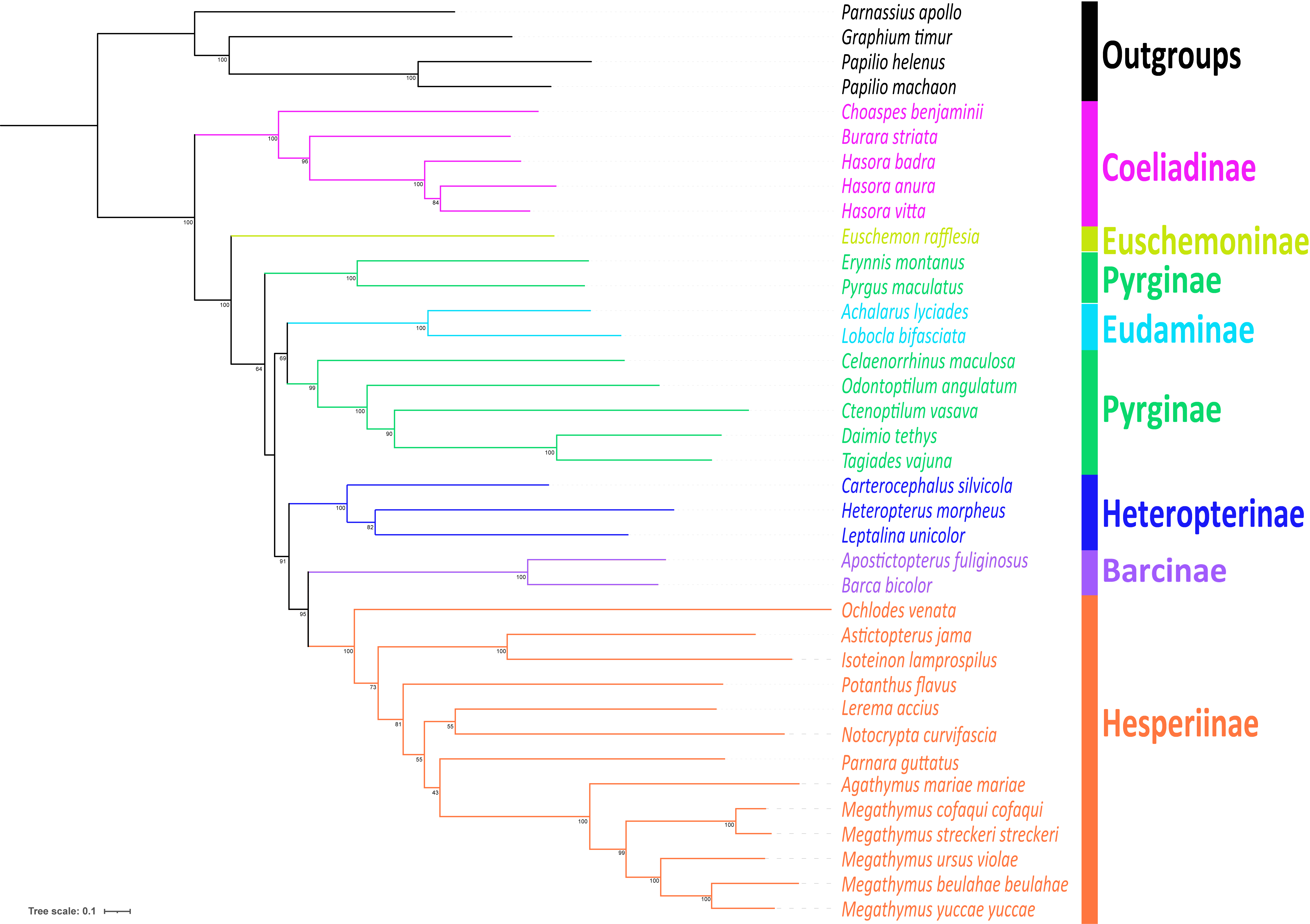

Supplement: Supplementary file 1 [file insects-12-00348-s001.zip › Fig. S3.tif]

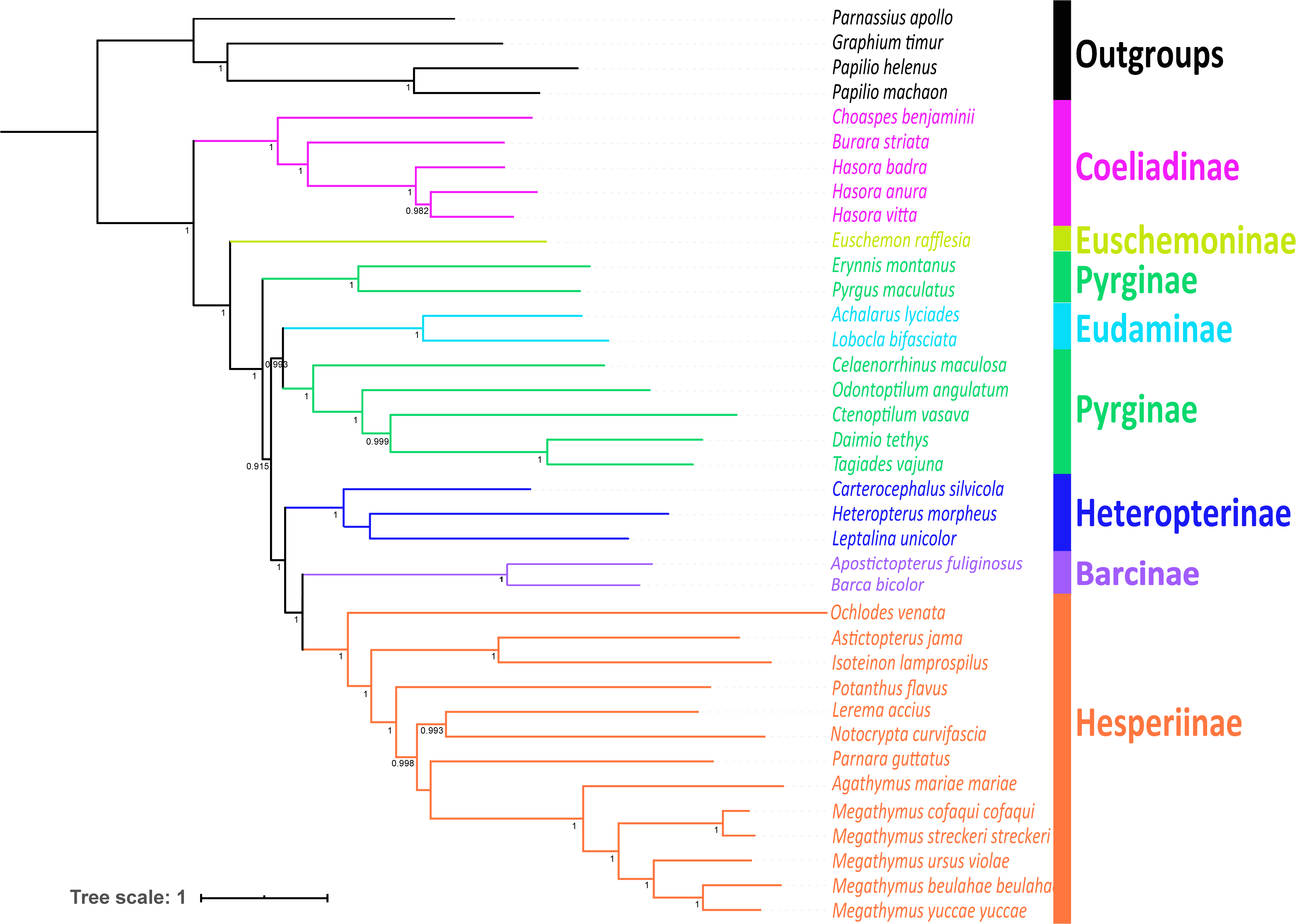

Supplement: Supplementary file 1 [file insects-12-00348-s001.zip › Fig. S4.tif]

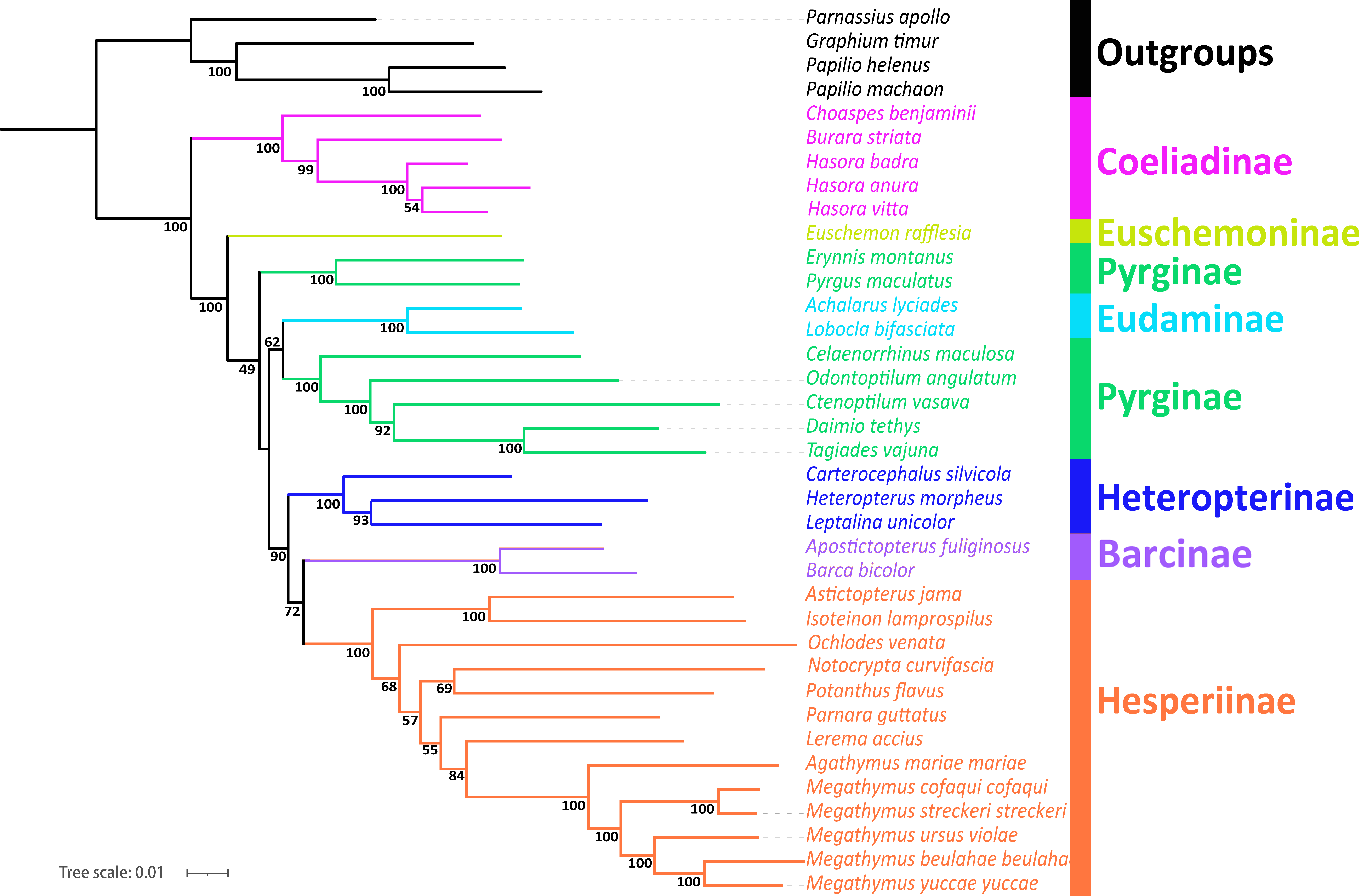

Supplement: Supplementary file 1 [file insects-12-00348-s001.zip › Fig.S5.tif]

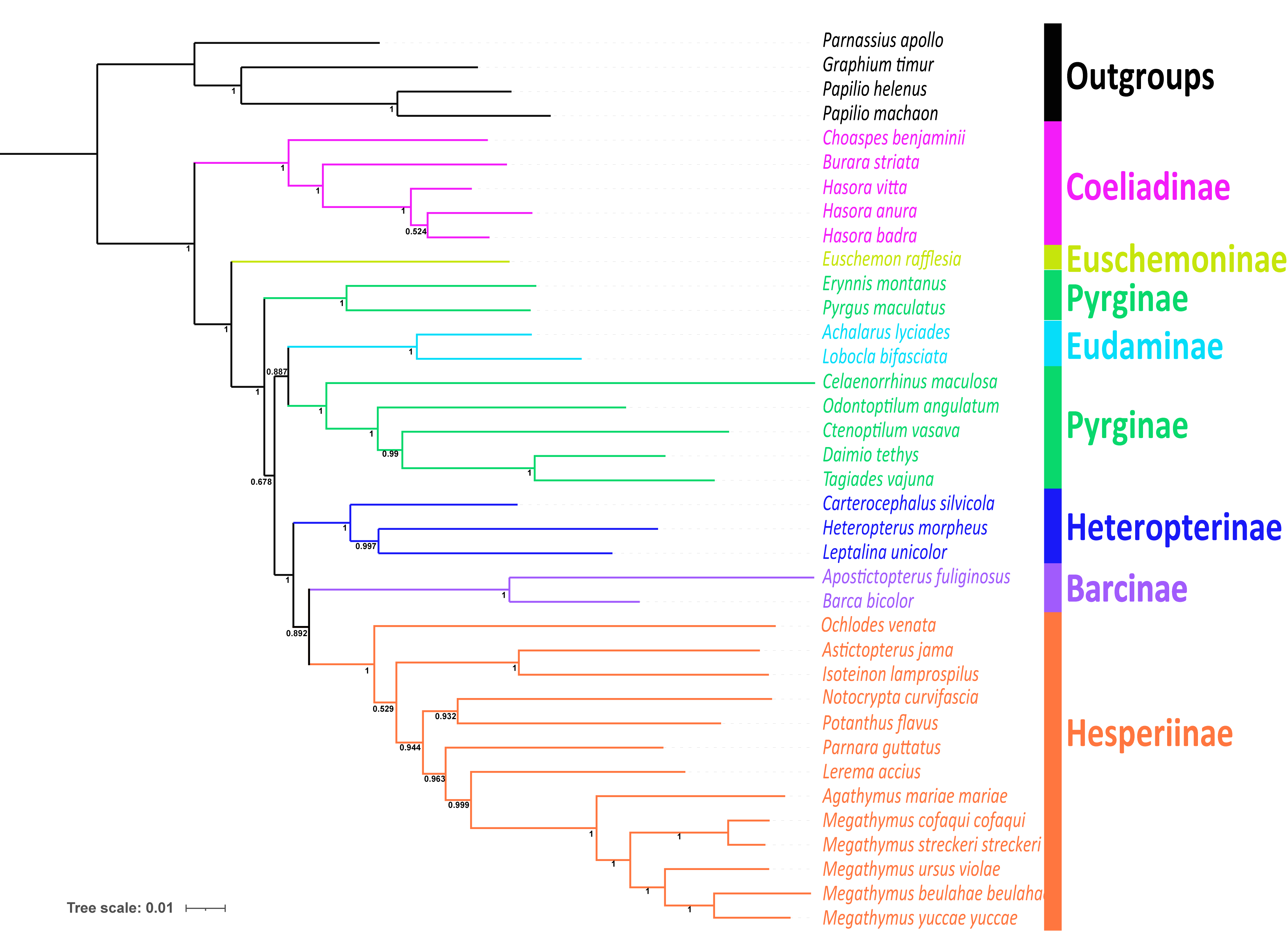

Supplement: Supplementary file 1 [file insects-12-00348-s001.zip › Fig.S6.tif]
